# Lipoma arborescens of the elbow

**DOI:** 10.4103/0971-3026.59755

**Published:** 2010-02

**Authors:** K Ranganath, Ganesh B Rao, Namitha

**Affiliations:** Department of Radiodiagnosis, RAGAVS Diagnostic and Research Centre Pvt Ltd, Sadguru Complex, No.14, 27^th^ Cross, 4^th^ Block West, Jayanagar, Bangalore - 560 011, India

**Keywords:** Elbow, Lipoma arborescens, MRI, synovium

## Abstract

Lipoma arborescens (LA) is a rare non-neoplastic intra-articular lesion that most commonly affects the knee joint, especially the suprapatellar bursa. It rarely affects the elbow joint. There are a few reports of involvement of the bicipital radial bursa. We report a case of LA, with characteristic MRI features, affecting the elbow joint in a young male.

## Introduction

Lipoma arborescens (LA) is a rare non-neoplastic intra-articular lesion that most commonly affects the knee joint, especially the suprapatellar bursa.[1] It rarely affects the elbow joint with a few reported cases of involvement of the bicipital radial bursa.[2] We report a case of LA, with characteristic MRI features, affecting the elbow joint in a young male.

## Case Report

**Figure 1 (a,b):**
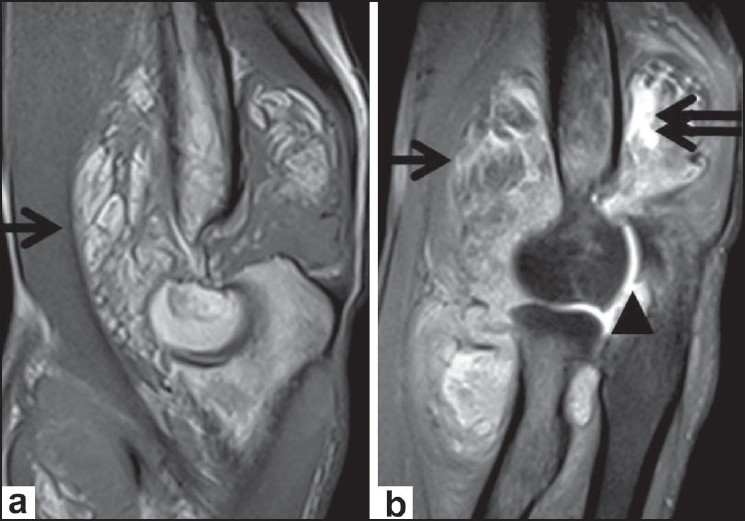
Sagittal proton density weighted (a) and proton-density, fat-suppressed (b) images of the elbow show synovial proliferation with fat signal intensity (arrow in a), with suppression (arrow in b). The joint effusion is noted (double arrow in b). Humero-radial and humero-ulnar osteophytosis (arrowhead) is noted

**Figure 2 (a,b):**
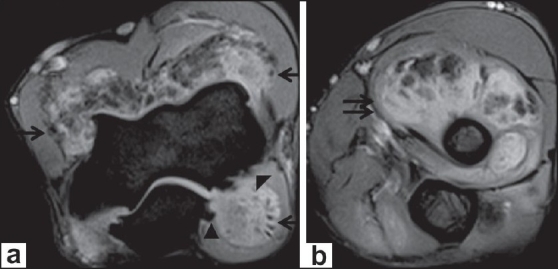
Axial T2W gradient images show synovial hypertrophy (arrows) with joint effusion, also extending into the bicipital radial bursa (double arrows) with marginal osteophytes involving the humerus and ulna (arrowheads)

**Figure 3 (a,b):**
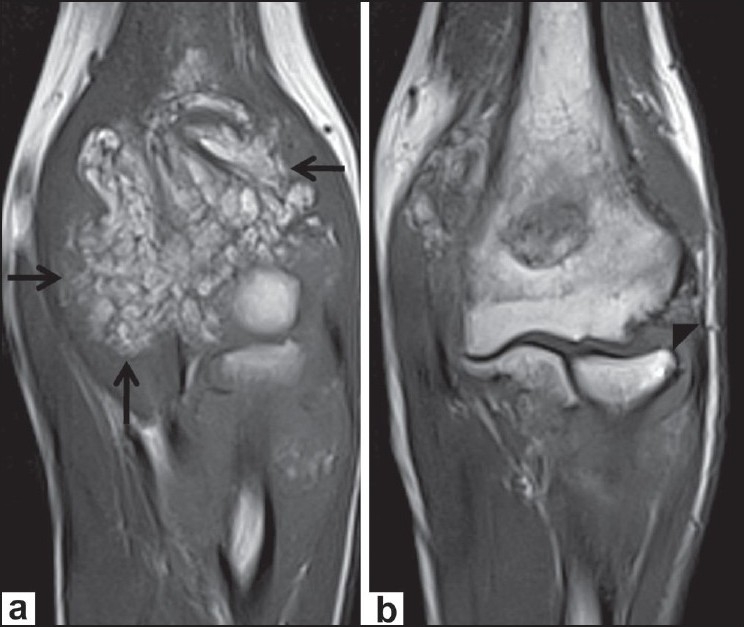
Coronal T1W images demonstrate fatty synovium (arrows) and marginal erosions involving the lateral epicondyle of the humerus (arrowhead)

**Figure 4:**
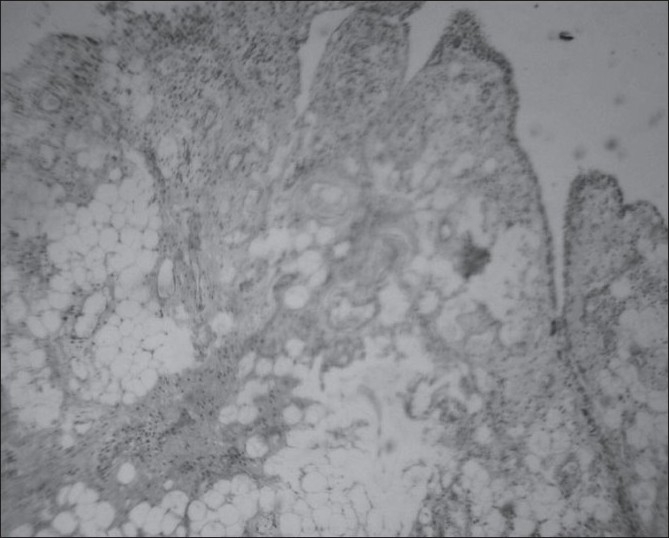
Photomicrograph of the synovial tissue (H-E stain) shows villous synovial hypertrophy with superficial synovial cell lining (arrow), subjacent chronic inflammatory cells and abundant subsynovial mature adipocytes giving a vacuolated appearance (arrowheads).

A 22-year-old male with complaints of gradual swelling of the left elbow for 3 years and pain for 1 year was referred for MRI of the left elbow. He had no past history of trauma or other joint problems. Laboratory investigations for rheumatoid arthritis factor and C-reactive protein were negative. MRI of the left elbow showed gross distension of the joint capsule due to a frond-like hypertrophy of the synovial tissue with signal intensity similar to that of fat on all sequences with an associated, large, joint effusion, which was best appreciated on the short tau inversion recovery (STIR) images [Figure 1]. The diffuse fatty synovial hypertrophy involved not only the radiocapitellar and humeroulnar joints but also the superior radioulnar articulation and the bicipital radial bursa [Figures 1 and 2]. Marginal erosions of the lateral epicondyle of the humerus and head of the radius were noted [Figure 3]. In addition, degenerative osteoarthritic changes were also noted involving the elbow joint, with thinning of the hyaline articular cartilage, narrowing of the joint space, and marginal osteophytosis [Figures 1 and 2]. Our diagnosis was primary LA of the left elbow joint associated with chronic synovitis and osteoarthritis. This was confirmed by excision biopsy, which revealed hypertrophied synovium with chronic synovial inflammation and underlying adipose tissue (diffuse subsynovial lipoma), consistent with the MRI findings [Figure 4].

## Discussion

LA is a rare non-neoplastic intra-articular lesion, most commonly affecting the knee joint (especially the suprapatellar bursa) and rarely occurring in other synovial joints, including the hip, ankle, shoulder, and wrist. It rarely affects the elbow joint. LA can uncommonly affect the synovial sheaths of tendons.[2,3]

LA can affect people of any age and has been reported in patients from 9 to 66 years of age.[4] It shows a slightly greater predilection for males. It can arise *de novo* or it may be a secondary reactive process associated with degenerative joint disease, chronic rheumatoid arthritis, or prior trauma.[3,4] It usually presents as a painless boggy joint swelling or recurrent effusion, which is usually monoarticular, but may also occur bilaterally.[4] LA (*arbor* means tree in Latin) is characterized by frond-like synovial proliferation, with replacement of the subsynovial tissue by mature fat cells, giving rise to villous synovial proliferation, which is the reason for the synonym ‘villous lipomatous proliferation of the synovium.’[4] It is also known as diffuse lipoma of the joint to distinguish it from a focal intra-articular lipomatous mass and is characterized histologically by subsynovial infiltration by mature fat cells (adipocytes) with interspersed chronic inflammatory cells.[4]

LA has a characteristic appearance on MRI.[1–3] There is frond-like synovial hypertrophy. The signal intensity of this synovial tissue is similar to that of subcutaneous fat on all sequences, being adequately suppressed on the fat-suppressed sequences. It is associated with joint effusion and secondary changes such as osteoarthritis and synovial cysts. Bone erosions secondary to chronic synovial hypertrophy may also be seen. The fatty synovial tissue does not enhance after administration of intravenous gadolinium. The other MRI features of LA include chemical shift artifacts at the interfaces of the lesion, effusion and the absence of a magnetic susceptibility effect, which may indicate the presence of hemosiderin and calcifications.[1,3,4]

Plain radiographs and CT scan may demonstrate radiolucent areas and fat density, respectively, within the intra-articular soft tissue mass.[3,4] They may also show osteoarthritic changes. USG demonstrates villous synovial hypertrophy, which appears uniformly hyperechoic against a background of effusion.[3] The synovial projections usually show wavelike motion with dynamic compression and manipulation.

Synovectomy is the treatment of choice for LA and recurrences are uncommon.[1,3]

To conclude, LA has characteristic features on MRI, which help to rule out other intra-articular synovial lesions like focal synovial lipoma, rheumatoid arthritis, pigmented villonodular synovitis, synovial chondromatosis, and synovial hemangioma.[1,4]
